# Protein biomarkers discriminate *Leishmania major*-infected and non-infected individuals in areas endemic for cutaneous leishmaniasis

**DOI:** 10.1186/s12879-016-1458-6

**Published:** 2016-03-24

**Authors:** Wafa Kammoun-Rebai, Ikbel Naouar, Valentina Libri, Matthew Albert, Hechmi Louzir, Amel Meddeb-Garnaoui, Darragh Duffy

**Affiliations:** Laboratory of Medical Parasitology, Biotechnologies and Biomolecules, Institut Pasteur de Tunis, Tunis, Tunisia; University of Tunis El Manar, Tunis, 1068 Tunisia; Laboratory of Transmission Control and Immunobiology of Infection, Institut Pasteur de Tunis, Tunis, Tunisia; Faculty of Medicine, Tunis, Tunisia; Center for Human Immunology, Institut Pasteur, Paris, France; Department of Immunology, Laboratory of Dendritic Cell Immunobiology, Institut Pasteur, Paris, France; Inserm U818, Paris, France

**Keywords:** Protein biomarkers, Leishmaniasis, Asymptomatic infection

## Abstract

**Background:**

A successful host immune response to infection is dependent upon both innate and adaptive immune effector mechanisms. Cutaneous leishmaniasis results in an adaptive Th1 CD4^+^ T cell response that efficiently clears the parasite, but may also result in scaring. However the role of innate mechanisms during parasite clearance remains less well defined.

**Methods:**

We examined a unique cohort of individuals, living in a *Leishmania major* endemic region, that were stratified among 3 distinct clinical groups in a cross-sectional study. Specifically, patients were classified either as healed (*n* = 17), asymptomatic (23), or naïve to infection (18) based upon the classical Leishmanin Skin Test (LST) and the presence or absence of scars. Utilizing a multiplexed immunoassay approach we characterized the induced cytokine and chemokine response to *L. major.*

**Results:**

A subset of innate immune molecules was induced in all groups. By contrast, T cell-associated cytokines were largely induced in exposed groups as compared to *L. major*-infection naïve individuals. Two exceptions were IL-17A and IL-12p70, induced and not induced, respectively, in naïve individuals. In addition, GM-CSF was more strongly induced in healed patients as compared to the other two groups. Surprisingly an IL-13 response was the best cytokine for classifying previously infected donors.

**Conclusions:**

Exploratory data analysis, utilizing principle component analysis (PCA), revealed distinct patient clusters of the healed and naïve groups based on the most differentially induced proteins. Asymptomatic previously infected individuals were more difficult to assign to a particular cluster based on these induced proteins. Analysis of these proteins may enable the identification of biomarkers associated with disease, leading to a better understanding of the protective mechanisms of immune response against leishmaniasis.

**Electronic supplementary material:**

The online version of this article (doi:10.1186/s12879-016-1458-6) contains supplementary material, which is available to authorized users.

## Background

Leishmaniasis is a parasitic disease endemic in 98 countries with more than 350 million people at risk worldwide [1], and approximately 1.3 million new cases and 20 000 to 30 000 deaths occur annually [2]. Zoonotic cutaneous leishmaniasis (ZCL), caused by *Leishmania major*, is endemic in Tunisia, since epidemic emergence in 1982 in Kairouan and expansion to the center and the south [3]. *L. major* is transmitted by the sand fly vector, Phlebotomus papatasi, and transmission is greatest from May to September. Infected humans can develop disease (ZCL lesions) between October and May [4]. Infected sand fly bites can lead to the development of cutaneous leishmaniasis, possibly resulting in scaring and a parasite-specific delayed-type hypersensitivity response as assessed by a positive Leishmanin Skin Test (LST) reaction. However this description is an oversimplification of the clinical scenario observed in exposed individuals. Field studies suggest that asymptomatic infection with *L. major* may occur in endemic areas but the extent of this phenomenon has not been fully evaluated [5].

Anti-*Leishmania* immunity is mediated by innate and adaptive immunity, with the CD4^+^ T cell subset crucial for resistance. Activated macrophages play a pivotal role in *Leishmania* infection through effective clearance of all forms of leishmaniasis [6]. Phagocytosis of parasites by macrophages induces the release of multiple chemoattractant factors, such as CXCL1, leading to recruitment of other innate immune cells [7]. Dendritic cells (DCs) also play a vital role in the production of IL-12 and type 1 IFNs, leading to activation of natural killer (NK) cells, IFN-γ production, and subsequent Th1 responses [8]. The magnitude of IL-12 production by infected DCs can critically affect the outcome of *L. major* infection [9] and can act in combination with IL-1α/β to influence Th1 T cell development and resistance to cutaneous infection [10]. The downstream result of IFN-γ and TNFα production is the generation of nitric oxide (NO) [11], a powerful cytostatic and cytotoxic molecule that plays a major role in killing many intracellular parasites, including *Leishmania*. However, *Leishmania* parasites possess the ability to modify the immune responses of their host to facilitate establishment of progressive infection. For example, mouse studies have shown that *Leishmania* induced IL-13 promotes disease [12–15], and numerous studies have identified a central role for IL-10 in susceptibility, immunopathology, and parasite persistence [16–18].

Stimulated PBMCs from patients are often used to assess immune function and evidence of previous or current infections. In this study we aimed to characterize the induced cytokine profile of individuals living in endemic areas of *L. major* transmission with different clinical features and a classically defined exposure history. In depth analysis of these immune profiles may allow the identification of biomarkers associated with disease outcome giving us a better understanding of the mechanisms behind protective human immune responses against leishmaniasis.

## Methods

### Study population and design

Our cross-sectional study population consisted of 58 individuals aged 7 to 18 years, living in an endemic area for ZCL due to *L. major* in the governorate of Sidi Bouzid and Kairouan in central Tunisia (Table 1). A physical and detailed skin examination was performed on each participant. The history of ZCL was assessed by the presence of typical scars, and blood samples were taken and the LST was administered. Individuals were subdivided according to the LST response (LST+ or LST-) and the presence of scars (SCAR+ or SCAR-). Within our study cohort there were 17 healed individuals (LST + SCAR+), 23 asymptomatic individuals (LST + SCAR-), and 18 naïve individuals (LST-SCAR-) (Table 1). All donors were recruited and samples taken during the months of April and May prior to the season of parasite transmission.

**Table 1:** Demographic and clinical features of the study population

	Cutaneous Leishmania (LST + SCAR+)	Asymptomatic (LST + SCAR-)	Naïve (LST-SCAR-)
- Donor numbers	17	23	18
- Age, mean ± SD (range), years	12.9 ± 3.5 (7–18)	13.2 ± 3.6 (7–18)	13.3 ± 4.4 (7–18)
- Zoonotic Cutaneous Leishmaniasis scars	yes	no	no
- Leishmanin Skin Test (LST) positive	yes	yes	no
- LST induration, mean ± SD (range), mm	12.3 ± 4.5 (5,5-21)	12.1 ± 5.6 (5–24,5)	0,1 ± 0,7 (0–3)

### Ethical statement

The study protocol was approved by the Institutional Review Board of the Pasteur Institute of Tunis. All subjects provided written informed consent for participation in the study and sample collection and analyses. For minors under the age of 18, written informed consent was obtained from a parent or guardian. The study was externally monitored for protocol agreement, data integrity, and protection of human subjects. For inclusion in our study we identified and selected individuals living in a *Leishmania* endemic area, aged 7 to 18 years old, who underwent a clinical examination to assess the presence or absence of ZCL history, and for whom an LST was performed. Exclusion criteria included immunodepression, pregnancy, and individuals who did not enable the LST interpretation in a follow up visit.

### Leishmanin skin test

LST was performed by intradermal injection of 100 μl of *L. major* suspension containing 5 x 10^6^ *L. major* promastigotes in 1 ml 0.5 % phenol saline. After 72 h, the induration was measured along 2 diameters by the ballpoint pen technique [19]. Induration with a diameter of 5 mm or more indicated a positive test [20–23].

### Parasite culture

*L. major* parasites (MHOM/TN/94/GLC94, zymodeme MON25) were cultured in Novy–Nicolle–McNeal medium at 26 °C and progressively adapted to RPMI 1640 medium (Sigma, St Louis, Mo) containing 2 mM L-glutamine (Sigma) 100 U/ml penicillin (Sigma), 100 mg/ml streptomycin (Sigma) and 10 % heat-inactivated fetal calf serum (FCS) (Invitrogen, CergyPontoise, France). Metacyclic promastigotes were isolated from day 6 stationary cultures by a discontinuous Ficoll gradient as previously described [24, 25].

### Isolation of human peripheral blood mononuclear cells (PBMCs) and cell stimulation

PBMCs were separated from heparinized blood samples using Ficoll/Hypaque (GE Healthcare, Uppsala) density gradient centrifugation. 1 x 10^6^ cells/ml were stimulated with live *L. major* parasite (3 parasites/cell), and with culture media as a negative control. Culture supernatants were collected after 48 hours and conserved at −80 °C until protein immunoassay testing.

### Multi-analyte protein immunoassays

Seventeen cytokines and chemokines were analysed by Luminex using an extended human Th1/Th2 12-plex (IFN-γ, IL-1β, IL-2, IL-4, IL-5, IL-6, IL-10, IL-12p70, IL-13, GM-CSF, TNFα, IL-18) and a custom made 6 plex (MIP-1α, IL-8, IL-17A, IFN-γ, MCP-1, M-CSF), according to the manufacturer’s instructions (Affymetrix, eBioscience). As IFN-γ was used as a positive control for infection, it was included in both Luminex assays and allowed for normalization between the two separately run assays. Results were expressed in pg/ml, and for data mining purposes, any samples with no measurable value were given a value that was half of the lowest detectable dose of the assays. IL-4 was not detected in any conditions so results are not presented, and MCP-1 was excluded due to technical reasons. All data is available in Additional file 1: Table S1.

### Statistical analysis

Wilcoxon matched-pairs tests were applied within clinical groups to test for a significant (*p* < 0.05) effect of parasite stimulation (GraphPad Prism). To test for significant differences (*p* < 0.05) between groups a Kruskal Wallis test with Dunn’s multiple test comparison was applied (GraphPad Prism). The sensitivity, specificity, positive and negative predictive values of the induced cytokine responses for IFN-γ, IL-2, IL-18, IL-12p70, and IL-13 for classifying LST+ (*n* = 40) individuals as compared to LST- (*n* = 18) with a 95 % confidence interval were calculated using GraphPad Prism. Principal component analysis (PCA) was applied for mutli-variate analyses on log transformed *L. major-*induced responses with *p* and *q* values reported following ANOVA testing (Qlucore Omics Explorer 3.1).

## Results

### Characterization of the adaptive immune response in LST+ individuals

**Fig. 1:**
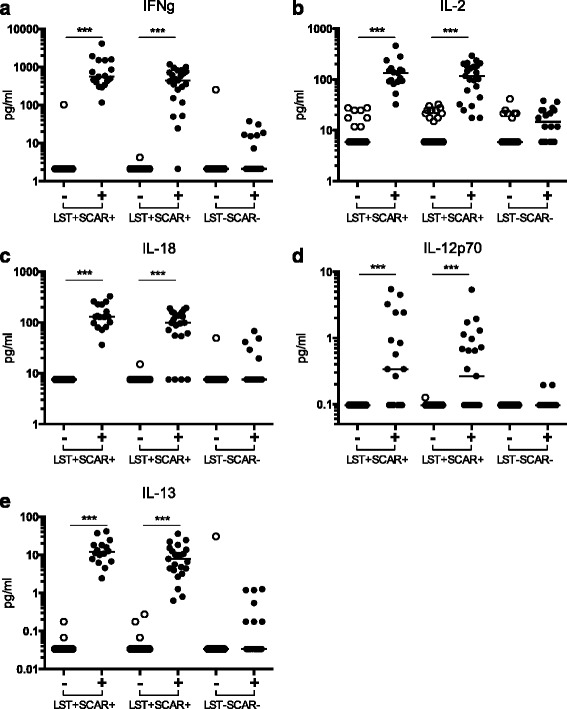
Characterization of the adaptive immune response in LST+ individuals. Secreted cytokines, (**a**) IFN-γ, (**b**) IL-2, (**c**) IL-18, (**d**) IL-12p70, and (**e**) IL-13 measured by Luminex assays following *Leishmania* parasite stimulation of PBMCs from donors subdivided into 3 clinical groups, according to the Leishmanin skin test response (LST+/−) and presence or absence of specific scars (SCAR+/−). (***) denotes *p* < 0.001 as determined by Wilcoxon paired *T* test, samples with no measurable value were given a value that was half of the lowest detectable dose of the assays)

The LST is a widely utilized method for evaluating an individual’s immune response to *Leishmania* infection. It is dependent on a delayed type hypersensitivity reaction, mediated by Th1 IFN-γ^+^ T cells, and indicates a previous infection and subsequent adaptive immune response. We confirmed a significant *Leishmania* specific IFN-γ response in the LST + SCAR+ and LST + SCAR- individuals, but not the LST-SCAR- naïve group following PBMC parasite stimulation (Fig. 1a). Utilizing Luminex multi-analyte assays we tested additional cytokines for potential differences among the cohort following PBMC parasite stimulation. Cytokines which showed a significant induction (Wilcoxon paired T test, *p* < 0.05) in the LST + SCAR+ and LST + SCAR- groups included IL-2, IL-18, IL-12p70, and IL-13 (Fig. 1b-e). Some LST-SCAR- donors secreted low amounts of IFN-γ, IL-2, IL-18, and IL-13 (Fig. 1a, b) but when analyzed as a group this response was not statistically significant. To test for potential utility in classifying patients we tested each cytokine for its sensitivity, specificity, positive and negative predictive values to characterise LST+ versus LST- patients (Table 2). Surprisingly IL-13 gave the best overall results with 100 % specificity and 86 % sensitivity, slightly superior than IFN-γ (92 % specificity and 86 % sensitivity). For IL-12p70 we had 92 % sensitivity though this result should be interpreted with caution as many of the values were close to the limit of detection of the assay. IFN-γ, IL-2 and IL-13 are all produced by T cells, but as IL-18, and IL-12p70 are produced by monocytes or dendritic cells we tested for other cytokines classically produced by these cell types across the 3 groups.

**Table 2:** The sensitivity, specificity, positive predictive values, and negative predictive values of the induced cytokine response for IFNγ, IL-2, IL-18, IL-12p70, and IL-13 for identifying LST+ (*n* = 40) individuals as compared to LST- (*n* = 16) with a 95 % confidence interval

	Sensitivity	Specificity	Positive predictive value	Negative predictive value
IFN-γ	0.86	0.92	0.97	0.66
IL-2	0.76	0.85	0.97	0.33
IL-18	0.87	0.76	0.9	0.72
IL-12p70	0.92	0.48	0.57	0.88
IL-13	0.86	1.00	1.00	0.66

### Shared induced innate immune responses across all 3 clinical groups

**Fig. 2:**
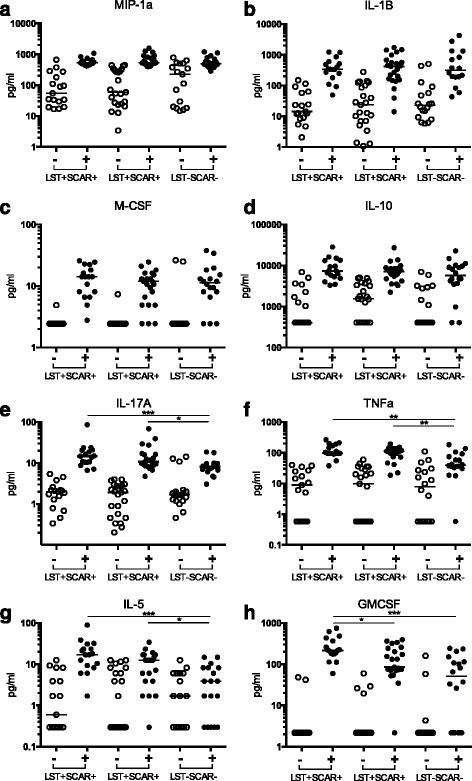
Shared induced innate immune responses across all 3 clinical groups. Secreted cytokines significantly induced (Wilcoxon paired T test, *p* < 0.05) (**a**) MIP-1α, (**b**) IL-1β, (**c**) M-CSF, (**d**) IL-10, (**e**) IL-17A, (**f**) TNFα, (**g**) IL-5, and (**h**) GMCSF measured by Luminex assays following *Leishmania* parasite stimulation of PBMCs from donors subdivided into 3 clinical groups, according to the Leishmanin skin test response (LST+/−) and presence or absence of specific scars (SCAR+/−). (*** denotes *p* < 0.001, ** *p* < 0.01, * *p* < 0.05 as determined by Kruskal Wallis test with Dunn’s multiple testing between the 3 groups, samples with no measurable value were given a value that was half of the lowest detectable dose of the assays)

In addition to the infection specific responses we observed a number of cytokines that were commonly induced in all groups following PBMC stimulation with *L. major* parasite, as determined by Wilcoxon paired T tests to non-stimulated controls (*p* <0.05) (Fig. 2). MIP-1α and IL-1β are two crucial innate immune mediators that were significantly induced in response to *L. major* parasites with little variation among the different clinical groups (Fig. 2a, b). However there was large variation (up to 2 log) between all individuals for the IL-1β, but not MIP-1α response. PBMCs from the three groups also commonly produced M-CSF and IL-10, though there were non-responders in all groups for M-CSF, and within the LST-SCAR- group for IL-10 (Fig. 2c, d). Of note the three IL-10 non-responders were high responders for M-CSF. Finally IL-17A, TNF-α, IL-5, and GMCSF were also induced in all groups following parasite stimulation (Fig. 2e-h). However IL-17A, TNF-α, and IL-5 were produced at significantly (*p* <0.05) higher levels in the previously infected donors compared to naïve individuals, (Fig. 2e-g). Finally, GMCSF was significantly (*p* <0.05) higher in LST + SCAR+ compared to both LST + SCAR- and LST-SCAR- individuals.

### Lack of induced response to *Leishmania* parasite for key inflammatory molecules

**Fig. 3:**
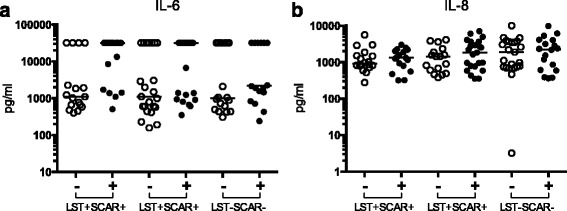
Lack of induced response to *Leishmania* parasite for key inflammatory molecules. Cytokines (**a**) IL-6, (**b**) IL-8 with no differences as measured by Luminex assays following parasite stimulation of PBMCs from donors subdivided into 3 clinical groups, according to the Leishmanin skin test response (LST+/−) and presence or absence of specific scars (SCAR+/−)

Two key inflammatory molecules, IL-6 and IL-8, did not show a significant difference following PBMC stimulation with the parasite in all 3 groups. IL-6 showed a clear bimodal distribution across all 3 groups, independent of parasite stimulation (Fig. 3a). However some individuals showed high levels of IL-6 either in the presence or absence of *ex vivo* parasite stimulation, suggesting that this cytokine may reflect another infectious or inflammatory process. IL-8, another key inflammatory modulator, also showed no significant induction following parasite stimulation across all 3 groups (Fig. 3b). This may reflect the PBMC stimulation where only monocytes but not neutrophils, of the main IL-8 producing cells, are present in this cell preparation. There was a trend towards higher overall IL-8 in the naïve donors, although this may be due to the higher variability in this group, as detected by a Bartlett’s test.

### Multivariate protein analysis reveals distinct patterns for LST + SCAR+ and LST-SCAR- groups

**Fig. 4:**
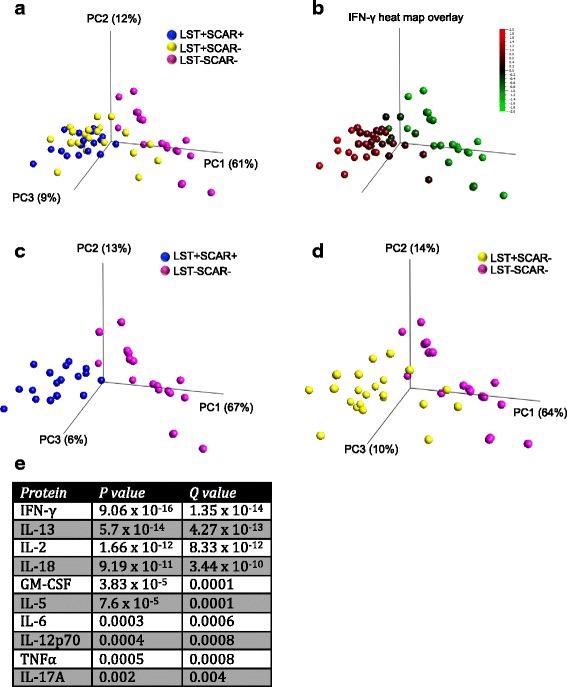
Multivariate protein analysis. Principal component analysis (PCA) of LST + SCAR+ (blue), LST + SCAR- (yellow), and LST-SCAR- (pink) donors (**a**) based on the 10 most significantly differential proteins as identified by multi ANOVA (q < 0.01), (**b**) heat map overlay of IFN-γ expression. PCA of (**c**) LST + SCAR+ (blue) and LST-SCAR- (pink) donors, (**d**) LST + SCAR- (yellow) and LST-SCAR- (pink) donors. Numbers represent percentage of variance captured by each principal component. (**e**) List of most differential proteins with p and q values as determined by multi ANOVA testing

To examine the complete response in a multivariate approach, we applied principle component analysis to all donors based on the 10 most differential proteins, as identified by a multi-group ANOVA (*p* <0.002, *q* <0.004). This revealed a clustering of the LST + SCAR+ group and the LST-SCAR- group mainly along the first principle component (Fig. 4a). The LST + SCAR- group clustered less clearly with some individuals clustering closer to the scarred group, and some to the naïve individuals. This indicates the heterogeneity within this group in terms of their induced immune response to the parasite. The first principle component (PC1), which comprised 61 % of the variance within the data set, was largely driven by IFN-γ and other T cell cytokines such as IL-2, IL-13, and IL-18. This is illustrated by a heat map overlay of IFN-γ protein levels on the PCA donor plot (Fig. 4b), and the list of proteins in order of significant differences as determined by ANOVA (Fig. 4e). PC2 which comprised 13 % of the variance, was mostly driven by MCP-1, IL-6, and GM-CSF suggesting a monocyte response. However this signature did not overlap with any of the clinical characteristics examined. PC3, which comprised 6 % of the total variance, was largely due to the IL-17A response which was higher in a subset of donors that were LST+. To discriminate the LST-SCAR- donors more clearly, we performed PCA in separate combinations with the other 2 groups. This confirmed a clear separation of uninfected donors from the LST + SCAR+ group, with a less clear distinction from the LST + SCAR- group (Fig. 4c-d).

## Discussion

The definition of immuno-pathological and immuno-protective mechanisms during human infection is difficult to achieve due to heterogeneity of both human genetic backgrounds and microbial populations. As such, human studies of the cellular immune responses during leishmaniasis are often descriptive or indirectly studied. In an attempt to counter some of these limitations, we examined a unique cohort of individuals living in a region endemic for ZCL, within which donors had 3 distinct clinical manifestations: exposed healed, exposed asymptomatic, and naïve, for whom we performed a multi-protein analysis following *ex vivo* parasite PBMC stimulation.

We confirmed the presence of a strong Th1 T cell response in previously infected individuals with a significant production of IFN-γ and IL-2. Experimental models of infection with *Leishmania* have been used to establish the importance of the Th1 immune response, whereas the Th2 response has been found to be associated with disease susceptibility [26]. However studies of human immunological responses have shown that the Th1/Th2 dichotomy shown in experimental animal models is not as evident in humans [26]. Higher IFN-γ production in individuals recovered from CL, and LST+ individuals without a history of CL, as compared to healthy controls has been previously reported by others [5, 27, 28]. In the LST+ donors, as well as detecting a strong IFN-γ response, we also observed a low but significant production of IL-13 [29]. This cytokine has been described as a susceptibility factor for *L. major* infection in mice [14], but data about IL-13 during human *Leishmania* infection are scarce. It was shown that IL-13 was the central mediator for maintaining Th2 development in human CL due to *L. guyanensis* by rendering specific cells unresponsive to IL-12 [30]. More recently, it was reported that IL-13 levels were similar in individuals with a history of self-healing CL due to *L. major* and healthy controls in response to soluble *Leishmania* antigen stimulation [31]. These differing results may be explained by substrain or species differences of *Leishmania* parasites and/or the different stimuli used (eg soluble antigen vs live parasites). Given the detection of IL-13, the lack of IL-4 in all cultures was surprising, as these cytokines share a common signalling pathway through the IL-4Rα chain and have previously been implicated in non-healing response to *L. major* infection in mice [32]. While this may reflect the kinetics of the stimulation system, or the sensitivity of the assays, it also highlights the complexity of cytokines and their often pleiotropic responses potentially determined by the local cellular microenvironment and overall immune status of the host.

Previously infected donors also showed a robust production of IL-18 and significant but low levels of IL-12p70, most likely produced by monocytes following parasite encounter. In the naïve group, we observed that a minority of donors secreted reduced levels of IFN-γ, IL-2, and IL-13, suggesting some low level immunity to the parasite. Interestingly the non-infected group secreted high amounts of IL-1β, without induction of IL-18. This was striking as these two cytokines are inflammasome-dependent. One possible explanation is that the initial production of IL-18 is dependent on the presence of adaptive cytokines only secreted in LST+ donors. A recent study showed that both cytokines were produced during *L. major* infection in mice [33], however the consequence of IL-18 induction for infection outcome is less clear. Some studies have shown a positive role for IL-18 through the amplification of Th1 responses, but others have shown a potential for biasing Th2 responses and infection susceptibility [33–35]. In contrast IL-1β has been shown to play a protective role through the downstream induction of nitric oxide by macrophages [36].

Notably, we observed significant IL-17A and TNF-α secretion by all donors, including naïve individuals who showed no evidence of IFN-γ production. Both of these cytokines have previously been associated with disease-induced immunopathology, with CD4^+^ T cells believed to be the main source during active disease [37, 38]. IL-17 is associated with inflammation and recruitment of neutrophils to the infection site during *L. major* infection in humans [39, 40] and as such may play a role in tissue damage at the site of infection.

The identification of IL-17-producing cells in PBMCs of naïve donors requires further study, as the major cell types previously identified to produce this cytokine are CD4^+^ T cells, and in some cases neutrophils. The role of IL-17 during *Leishmania* infection is controversial. IL-17 promoted progression of CL in *L. major*-infected mice via regulation of neutrophil recruitment [41, 42], whereas in *L. infantum*-infected mice it was shown to promote the control of parasite replication by acting synergistically with IFN-γ to potentiate NO production [43]. In humans, IL-17 is associated with inflammation and recruitment of neutrophils at the infection site during *L. braziliensis* infection [29, 30] whereas in VL it is associated with protection [44]. To our knowledge our study is the first to report the presence of an IL-17A response in individuals immune to *L. major.*

GM-CSF was produced at significantly higher levels in both infected groups compared to naïve donors. In addition it showed a strong positive correlation (>75 %, *p* < 0.0001) with both TNF-α and IL-5, which shared the same pattern of higher production in infected asymptomatic patients. This interesting finding of significantly higher levels of GM-CSF found in individuals with previous ZCL compared to both asymptomatic and naïve individuals, could suggest that individuals predisposed to higher production of GM-CSF may be at risk for developing clinical disease when infected with *Leishmania*. High levels of GM-CSF favour macrophage recruitment and differentiation, the main target population in which *Leishmania* has evolved strategies for efficient uptake and survival. These results are in agreement with a previous study demonstrating successful treatment of refractory CL with GM-CSF and antimonials [45].

Whether the differential expression of these cytokines influences the clinical manifestation of disease, or is a direct reflection of disease requires further study. However these results show promise for new diagnostic approaches based on the measurement of induced immune responses, as opposed to the crude LST test, which requires an invasive skin injection of parasite in phenol solution. The use of secreted IFN-γ as a biomarker to replace the LST test has already been proposed [46]. However the inclusion of additional protein biomarkers may improve diagnostic accuracy. For example, in our cohort IL-13 showed greater specificity than IFN-γ for the identification of LST+ patients. To fully capitalize on the application of multi-analyte protein testing in diagnostic settings new analytical approaches are required. As some of these methods, which include principal component analysis, have recently been used to identify potential biomarkers in HIV [47] and viral hepatitis [48], we applied this approach to our patient data set. While it did not reveal new findings per se, this approach illustrated the heterogeneous nature of the asymptomatically infected patients. In addition it confirmed the presence of an adaptive immune response that segregated LST+ from LST- patients, and an innate immune signature that did not help patient segregation. While the use of more classical statistical approaches identified potential biomarker combinations with improved diagnostic accuracy, larger validation studies, as well as more standardized methods of *ex vivo* stimulation [49], will be required to validate these findings. In addition, these new approaches may aid in the elucidation of the precise mechanisms and role of cytokines in leishmaniasis pathology.

## Conclusion

Our study provides new insights into leishmaniasis by showing distinct clusters of healed and naïve patient groups based on the most differentially induced proteins following *ex vivo* parasite stimulation. Asymptomatic individuals were more difficult to assign to a particular cluster based on these induced proteins. These proteins deserve further consideration to identify biomarkers associated with disease outcome.

## Additional file

Additional file 1: Table S1. Luminex protein data set. (XLSX 63 kb)

## Abbreviations

CL: cutaneous leishmaniasis
LST: leishmanin skin test
ZCL: Zoonotic Cutaneous Leishmaniasis
